# Docosahexaenoic acid-induced apoptosis is mediated by activation of mitogen-activated protein kinases in human cancer cells

**DOI:** 10.1186/1471-2407-14-481

**Published:** 2014-07-03

**Authors:** Soyeon Jeong, Kaipeng Jing, Nayeong Kim, Soyeon Shin, Soyeon Kim, Kyoung-Sub Song, Jun-Young Heo, Ji-Hoon Park, Kang-Sik Seo, Jeongsu Han, Tong Wu, Gi-Ryang Kweon, Seung-Kiel Park, Jong-Il Park, Kyu Lim

**Affiliations:** 1 Department of Biochemistry, School of Medicine, Chungnam National University, Daejeon 301-747, Korea; 2 Cancer Research Institute, School of Medicine, Chungnam National University, Daejeon 301-747, Korea; 3 Infection Signaling Network Research Center, School of Medicine, Chungnam National University, Daejeon 301-747, Korea; 4 Department of Pathology and Laboratory Medicine, Tulane University School of Medicine, New Orleans, LA 70112, USA

**Keywords:** Docosahexaenoic acid, Reactive oxygen species, Mitogen-activated protein kinases, Apoptosis, Cancer

## Abstract

**Background:**

The role of omega-3 polyunsaturated fatty acids (ω3-PUFAs) in cancer prevention has been demonstrated; however, the exact molecular mechanisms underlying the anticancer activity of ω3-PUFAs are not fully understood. Here, we investigated the relationship between the anticancer action of a specific ω3-PUFA docosahexaenoic acid (DHA), and the conventional mitogen-activated protein kinases (MAPKs) including extracellular signal-regulated kinase (ERK), c-JUN N-terminal kinase (JNK) and p38 whose dysregulation has been implicated in human cancers.

**Methods:**

MTT assays were carried out to determine cell viability of cancer cell lines (PA-1, H1299, D54MG and SiHa) from different origins. Apoptosis was confirmed by TUNEL staining, DNA fragmentation analysis and caspase activity assays. Activities of the conventional MAPKs were monitored by their phosphorylation levels using immunoblotting and immunocytochemistry analysis. Reactive oxygen species (ROS) production was measured by flow cytometry and microscopy using fluorescent probes for general ROS and mitochondrial superoxide.

**Results:**

DHA treatment decreased cell viability and induced apoptotic cell death in all four studied cell lines. DHA-induced apoptosis was coupled to the activation of the conventional MAPKs, and knockdown of ERK/JNK/p38 by small interfering RNAs reduced the apoptosis induced by DHA, indicating that the pro-apoptotic effect of DHA is mediated by MAPKs activation. Further study revealed that the DHA-induced MAPKs activation and apoptosis was associated with mitochondrial ROS overproduction and malfunction, and that ROS inhibition remarkably reversed these effects of DHA.

**Conclusion:**

Together, these results indicate that DHA-induced MAPKs activation is dependent on its capacity to provoke mitochondrial ROS generation, and accounts for its cytotoxic effect in human cancer cells.

## Background

Omega-3 polyunsaturated fatty acids (ω3-PUFAs) have the first double bond in the ω3 position (third carbon from the methyl end of the carbon chain) and are considered essential fatty acids because they cannot be synthesized by mammals [1]. These PUFAs are able to regulate eicosanoid production [2], transcription events [3], formation of potent lipid peroxidation products [4], Wnt/β-catenin signaling [5,6], and autophagy [7]. Docosahexaenoic acid (DHA) and eicosapentaenoic acid (EPA) are the main long chain ω3-PUFAs, and their anticancer effects have been demonstrated, with DHA showing a stronger effect than EPA because of the higher degree of unsaturation of the DHA molecule [8].

Various cellular metabolic processes are associated with the generation of reactive oxygen species (ROS) including hydrogen peroxide (H_2_O_2_), superoxide anion, and hydroxyl radicals as chemically reactive molecules [9]. ROS regulate crucial cellular events, such as transcription factor activation, gene expression, and cell differentiation and proliferation [10]. In mammalian cells, an important source of ROS generation is the mitochondrial electron transport chain [11]. Overproduction of ROS induces cellular damage, such as the oxidation of cardiolipin in the mitochondrial membrane and a decrease in the mitochondrial membrane potential (MMP), which leads to apoptotic cell death [9,11].

ROS activate the mitogen-activated protein kinases (MAPKs) families, which regulate many cellular processes, including cell growth, proliferation, differentiation, survival, and death [12]. Mammals express at least three conventional MAPKs, extracellular signal-regulated kinase (ERK), c-JUN N-terminal kinase (JNK) and p38, and dysregulation of the conventional MAPKs is implicated in human cancers [13]. While JNK and p38 activation is related to apoptosis under environmental stress conditions, especially oxidant injury, the activation of ERK induced by mitogens, growth factors and cytokines is generally believed to trigger pro-survival signals [14]. However, recent studies suggest that ERK activation can also lead to apoptotic death of tumor cells in repsonse to various anticancer agents [15]. For example, cisplatin-induced apoptosis in human cancer cells has been attributed to ERK activation, and inhibition of ERK markedly attenuates the pro-apoptotic effect of cisplatin [16].

In the present study, we investigated the cell death mode induced by DHA in four cancer cell lines derived from different types of cancers, and explored the relationship between conventional MAPKs and the cytotoxic effect of DHA. Our results show that DHA induces apoptotic cell death via ROS-regulated MAPK activation. These results have important implications for the chemoprevention and treatment of human cancer using ω3-PUFAs.

## Methods

### Chemicals and antibodies

DHA (Cayman Chemical, Ann Arbor, MI, USA) and tetramethylrhodamine ethyl ester (TMRE, Invitrogen, Camarillo, CA, USA) dissolved in absolute ethanol, Dihydroethidium (DHE, Invitrogen), PD98059 (Calbiochem, Cambridge, UK), SP600125 (Calbiochem), SB600125 (Calbiochem) and MitoSOX Red (Invitrogen) dissolved in dimethyl sulfoxide (Sigma, ST Louis, MO, USA), N-acetyl-L-cystein (NAC, Sigma) dissolved in phosphate buffered saline and H_2_O_2_ (MERCK, Darmstadt, Germany) dissolved in distilled water were stored at -20°C before use.

The antibodies used and their sources are as follows. Caspase-3, JNK, p38, phospho-p38 (Thr180/Tyr182) and XIAP antibodies were purchased from Cell signaling Technology (Beverly, MA, USA); antibodies against PARP-1/2 (H-250), phospho-ERK (E-4), ERK1 (K-23), Survivin and actin (I-19)-R were from Santa Cruz (CA, USA); goat anti-rabbit and goat anti-mouse secondary antibodies were from Calbiochem; and phospho-JNK1&2 (pT183/pY185) antibodies and secondary antibodies (goat anti-rabbit and goat anti-mouse) conjugated with TRITC were from Invitrogen.

### Cell cultures and chemical treatment

Human ovarian cancer PA-1 cells, human lung cancer H1299 cells, and human cervical cancer SiHa cells were purchased from American Type Cell Culture Collection (Rockville, MD, USA). Human glioblastoma D54MG cells were provided by Dr. Binger (Duke University Medical Center, Durham, NC, USA). PA-1 cells were maintained in Minimum Essential Medium (MEM, GIBCO, Grand Island, NY, USA); H1299 and SiHa cells were maintained in Dulbecco’s Modified Eagle Medium (DMEM); and D54MG cells were maintained in RPMI 1640 medium (GIBCO). The media were supplemented with 10% heat-inactivated fetal bovin serum (FBS, GIBCO), penicillin and streptomycin. The cells were cultured in a humidified 5% CO_2_ atmosphere at 37°C.

Cells grown to 70% confluency were switched into serum-free media, and the cultures (H1299, D54MG and SiHa) were allowed to expand for 24 h before giving any treatment. For PA-1 cells, the serum-free culture condition was used at 12 h, as an incubation time longer than 12 h resulted in slight loss of cell viability (data not shown).

### Cell viability assay

Cells were plated onto 96-well plates at seeding densities of 6.5 × 10^3^ cells per well for PA-1, H1299 and SiHa cells and 7 × 10^3^ cells per well for D54MG cells. The cell viability after treatment with appropriate agents was measured using Thiazolyl Blue Tetrazolium Bromide (MTT, Sigma) as previously described [17]. Concentrations of DHA that produced 50% inhibition in cell survival (IC_50_) following a 24 h exposure, were manually derived from dose–response curves generated by the Microsoft Excel 2010 edition.

### Measurement of oxygen consumption rate (OCR)

Cellular oxygen consumption was measured using a Seahorse bioscience XF24 analyzer (Seahorse Bioscience Inc., North Billerica, MA, USA) in 24-well plates at 37°C, with correction for positional temperature variations adjusted from four empty wells evenly distributed within the plate. PA-1 cells were seeded at 4 × 10^4^ cells per well 18 h prior to the analysis, and each experimental condition was performed on 4 biological replicates. Immediately before the measurement, cells were switched to 1% FBS contained MEM for 4 h. Then cells were washed and 590 μL of non-buffered media (sodium bicarbonate free, pH 7.4 DMEM) was added to each well. After 15 min equilibration period, three successive 2 min measurements were performed at 3 min intervals with inter-measurement mixing to homogenize oxygen concentration in the medium and each condition was measured in independent walls. Concentrated compounds (10X) were injected into each well using the internal injector of the cartridge and three successive 2 min measurements were performed at 3 min intervals with inter-measurement mixing.

### Western blot, immunocytochemistry and apoptosis assays

Western blot, immunocytochemistry and apoptosis assays were done as described previously in reference [7].

### Determination of intracellular ROS and MMP

ROS production was measured using fluorescent probes DHE, and MitoSOX. Cells seeded onto 6-well plates were first stained with either DHE (10 μM) or MitoSOX (5 μM) in Hanks’ balanced salt solution (HBSS) for 30 min (15 min in case of MitoSOX) at 37°C. After washing away unbound probes, cells were switched into serum-free media, pretreated with or without 5 mM of NAC for 1 h and exposed to DHA for 4 h. Direct imaging of ROS in probe-stained cells was performed using a fluorescence microscope (Olympus iX70, Japan), and images were captured with a DP Controller software. All images were taken under identical exposure conditions to assess the intensity of the probe fluorescence accurately. Alternatively, the probe-stained cells were detached with trypsin-EDTA, washed and fluorescence intensity was measured within 60 min by flow cytometry. For each sample, at least 10,000 events were acquired and analyzed using the BD FACS-Calibur (BD Bioscience, San Diego, CA, USA). MMP levels were evaluated using fluorescent probes, TMRE. In brief, cells were stained with TMRE at a concentration of 25 nM for 15 min at 37°C in HBSS, washed twice, and then preincubated with or without 5 mM of NAC for 1 h in serum-free media before DHA exposure. After incubation with DHA for 4 h, the fluorescence of the cells stained with TMRE was monitored by flow cytometry as described above.

### Small interfering RNAs (siRNAs)

siRNAs for human ERK1/2, JNK1/2 and p38 were purchased from Bioneer (Daejeon, Korea). For transfection, 25 nM siRNAs were added to 9 × 10^5^ cells in a 100 mm dish using Lipofectamine RNAiMAX (Invitrogen) as recommended by the vendor. Control cells were transfected with a negative control siRNA with no known mRNA target (5’-ACG UGA CAC GUU CGG AGA AUU-3’) designed by Bioneer. After 18 h of transfection, cells were switched into serum-free media for 24 h (12 h in case of PA-1 cells) and then treated with DHA. The siRNAs sequences used were: *ERK1* (5′-GAC CGG AUG UUA ACC UUU A-3′), *ERK2* (5′-CCA AAG CUC UGG ACU UAU-U-3′), *JNK1* (5′-CUG GUA UGA UCC UUC UGA A-3′), *JNK2* (5′-CUG UAA CUG UUG AGA UGU A-3′) and *p38* (5′-CAA AUU CUC CGA GGU CUA A -3′)*.*

### Statistical analysis

Student’s t test was performed for statistical analyses. In all analyses, the level of statistical significance was more than the 95% confidence level (*P* < 0.05). *** means *P* < 0.001.

## Results

### DHA inhibits cell viability and induces apoptosis in human cancer cells

**Figure 1:**
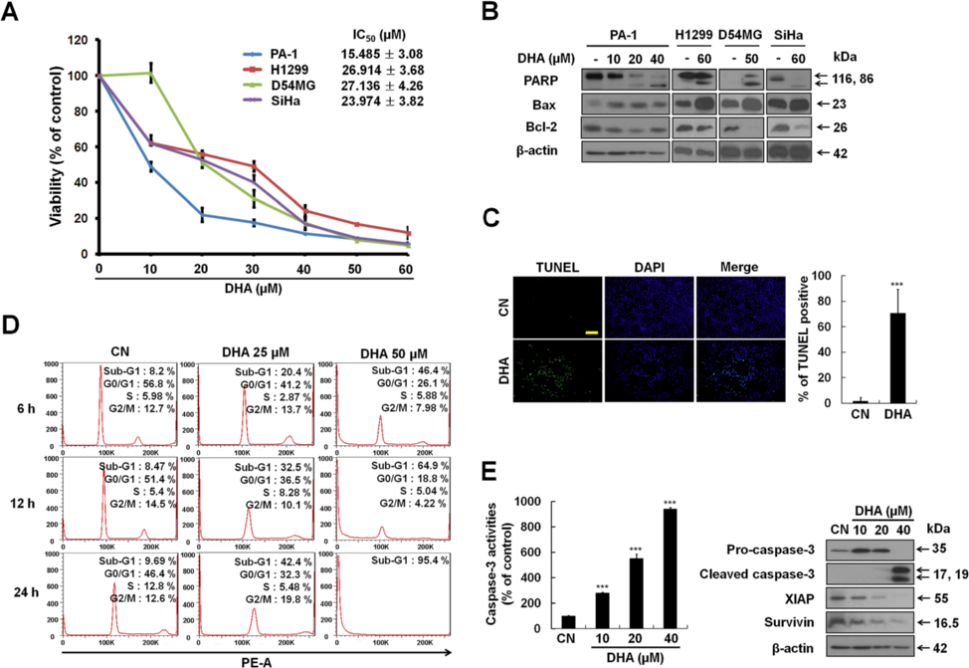
**DHA reduces viability and induces apoptotic death of human cancer cells. (A)** DHA dose-dependently decreases the viability of PA-1, H1299, D54MG and SiHa cells. Cells were treated with the indicated doses of DHA for 24 h, and the cell viability was measured with MTT assays as described in the Materials and Methods. Each bar represents the mean of three determinations repeated in three separate experiments. **(B)** DHA induces apoptosis. Human cancer cells were incubated with DHA at the indicated doses, and cells were harvested and western blot analysis was performed using PARP, Bax, Bcl-2 and actin antibodies. **(C)** D54MG cells were incubated for 6, 12, 24 h with indicated doses of DHA, and Sub-G1 DNA contents were evaluated by flow cytometric analysis. Samples were analyzed using FlowJo software. **(D)** DHA increases the number of TUNEL positive PA-1 cells. Cells were plated on coverslips, and incubated with or without DHA for 6 h. The cells were stained with DeadEnd Fluorometric TUNEL system. Left, the results are shown as a microscopy image. DNA was counterstained with DAPI (scale bar, 200 μm). Right, the percentage of TUNEL positive cells treated with or without DHA was calculated relative to the total number of DAPI-stained nuclei. TUNEL positive cells were counted in three different fields and averaged. ***, *P* < 0.001. **(E)** Increases in caspase-3 activities and caspase-3 cleavage formation by DHA. PA-1 cells were treated with various concentrations of DHA for 12 h and lysed. Left, Caspase-3 activity was determined using the fluorogenic substrate DEVD-AFC. Values are mean ± SEM (n = 5). ***, *P* < 0.001. Right, western blot analysis of cleaved caspase-3, XIAP and Survivin. Equal loading of protein lysate was confirmed using an anti-actin antibody.

To examine the effect of DHA on the growth of human cancer cells, PA-1, H1299, D54MG and SiHa cells originating from ovarian, lung, brain and cervical tumors were cultured with increasing concentrations (0–60 μM) of DHA for up to 48 h, and the cell viability was measured by MTT assays. DHA reduced cell viability in a dose- and time-dependent manner in all four cell lines studied (Additional file 1: Figure S1A). Figure 1A shows the viability and IC_50_ values of the cells after multiple doses of DHA exposure for 24 h. Four cell lines exhibited different sensitivity to DHA, and the IC_50_ values for PA-1, H1299, D54MG and SiHa cells were 15.485 ± 3.08, 26.914 ± 3.68, 27.136 ± 4.26 and 23.974 ± 3.82 μM, respectively.

To determine whether the observed reduction in cell viability was caused by apoptosis, DHA-treated cells were first examined for cleavage of the apoptosis marker PARP and expression levels of Bcl-2 family proteins, which play critical roles in the apoptotic process [18]. While DHA increased the expression levels of cleaved PARP and pro-apoptotic Bax, it attenuated the expression level of anti-apoptotic Bcl-2 (Figure 1B). In addition, DHA induced the formation of DNA strand breaks/hypodipliod nuclei (a typical characteristic of apoptotic cells [18]) as evidenced by an increased number of TUNEL positive cells (Figure 1C) and the cells with Sub-G1 DNA content (Figure 1D and Additional file 2: Figure S2). Notably, the elevated Sub-G1 population was directly paralleled by diminished proportions of D54MG (Figure 1D) and PA-1 cells (Additional file 2: Figure S2A) in each cell-cycle phase. However, a transient increase in the cell populations in G2/M phase was detected 6 h after 30 μM DHA treatment in H1299 and SiHa cell lines (Additional file 2: Figure S2B-S2C), implying that DHA may also interfere with cell-cycle distribution. Next, we measured the activity and cleavage formation of caspase-3, an executor caspase that is activated through both intrinsic and extrinsic apoptosis pathways [18], using PA-1 cells. Our results showed that DHA dose-dependently activated caspase-3 (Figure 1E, left), and upregulated the level of cleaved caspase-3 (Figure 1E, right and Additional file 1: Figure S1B). It is known that the inhibitor of apoptosis proteins (IAPs) are able to suppress apoptosis by inhibiting caspase-3 [19]. We thus also determined the effect of DHA on expression of two well-documented IAP family members, Survivin and XIAP (Figure 1E, right). Levels of Survivin and XIAP were decreased markedly after DHA treatment. These results indicate that DHA induces apoptosis, which contributes to the inhibitory effect of DHA on cancer cell growth.

### *DHA leads to* MAPK activation

**Figure 2:**
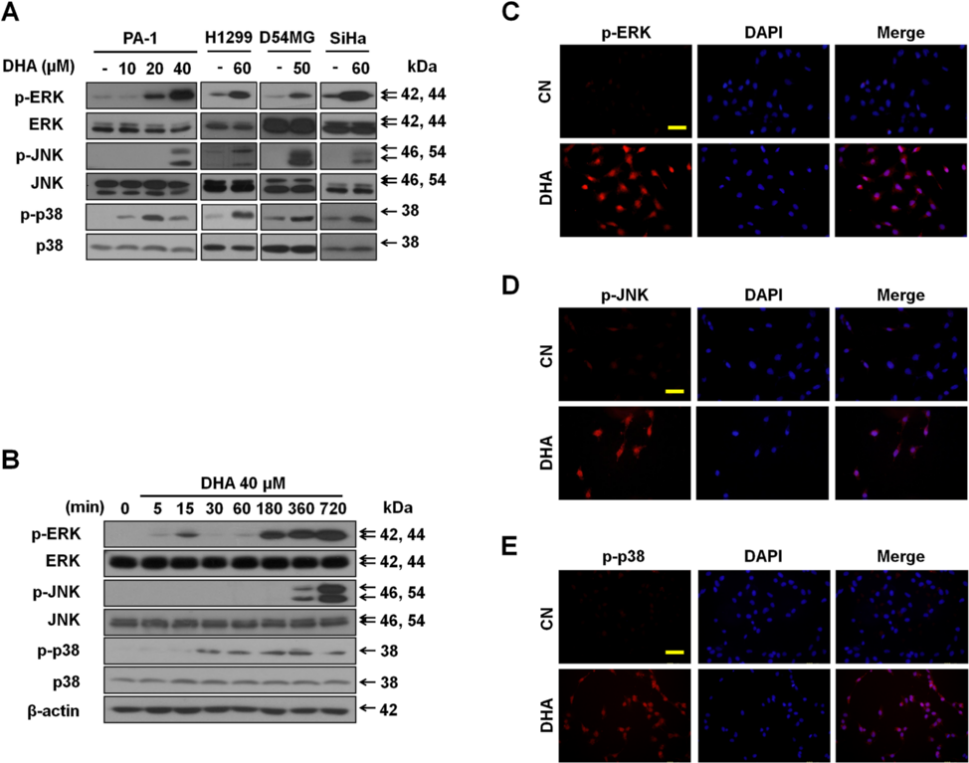
**DHA activates MAPKs. (A)** DHA induces MAPKs activation. PA-1, H1299, D54MG and SiHa cell lines were treated with the indicated doses of DHA for and 24 h (12 h in case of PA-1 cells). Then, protein lysates were separated and immunoblotted with antibodies against conventional MAPKs. **(B)** Expression patterns of conventional MAPKs in response to DHA over time. PA-1 cells treated with 40 μM DHA for the indicated time periods were subjected to immunoblotting for MAPKs. **(C-E)** Nuclear accumulation of phospho-ERK, -JNK, and -p38 in PA-1 cells after DHA exposure. PA-1 cancer cells were incubated for 6 h with or without 40 μM DHA. Then, cells were stained with antibodies against phospho-ERK **(C)**, phospho-JNK **(D)** and phospho-p38 **(E)** and analyzed by immunoflurescence. Scale bars, 50 μm.

Conventional MAPKs play important roles during cancer progression, and have been shown to be activated during the apoptotic death of tumor cells in response to various cellular stresses [13-15,20]. To gain insights into the mechanisms by which DHA induces apoptosis in cancer cells, we first investigated whether DHA treatment resulted in the activation of conventional MAPKs. Immunoblotting revealed that DHA, used at concentarions triggering apoptosis, remarkably elevated the phosphorylation levels of ERK/JNK/p38 in all four cell lines (Figure 2A). The phosphorylation of ERK and p38 became apparent at relatively earlier time points tested (0.5-3 h) following treatment of PA-1 cells with 40 μM DHA (Figure 2B). Additionally, a rapid and transient increase in ERK phosphorylation was observed after 15 min of treatment, which is in line with ERK activation being an indicator of stress [21]. Because MAPK signaling involves the activation of transcription factors [14], immunocytochemistry assays were performed to determine whether the activation of MAPKs was accompanied by their accumulation in nuclei. Figure 2C-E show that the fluorescence intensity of phospho-ERK, -JNK, and -p38 was increased in DHA-treated cells. Furthermore, DHA also increased the number of cells with nuclear staining for these phosphorylated MAPKs. These data together indicate that DHA activates the conventional MAPKs in cancer cells.

### *DHA induces mitochondrial ROS* production

**Figure 3:**
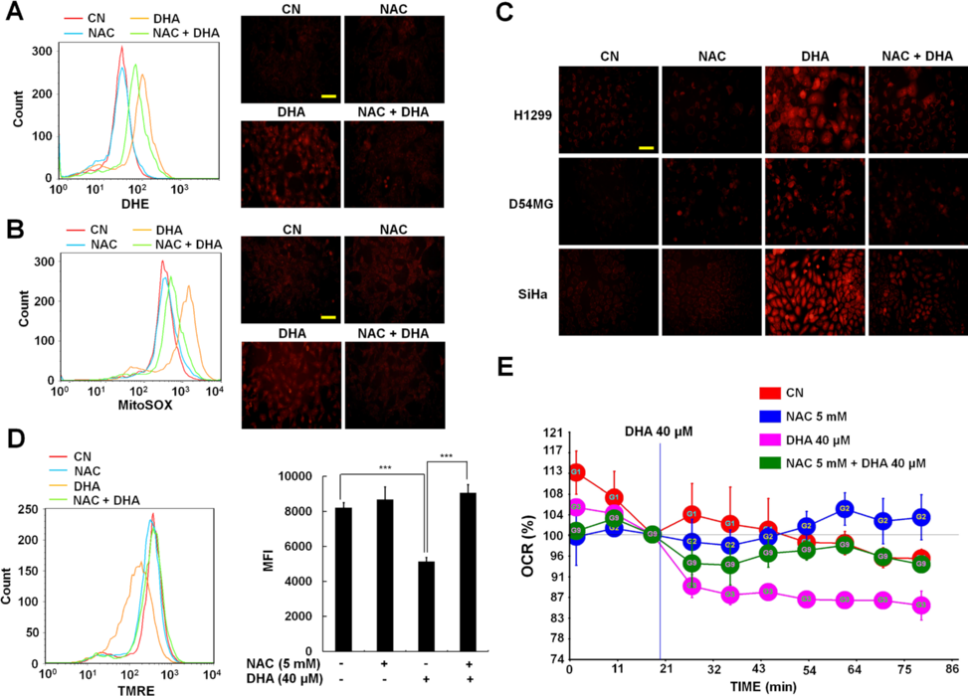
**DHA induces mitochondrial ROS overproduction and mitochondrial dysfunction. (A-B)** PA-1 cells were incubated for 1 h with or without 5 mM NAC before exposure to 40 μM DHA for 4 h. Intracellular superoxide and mitochondrial superoxide levels were detected using DHE **(A)** or MitoSOX **(B)** probes under a fluorescence microscope (right) or by flow cytometry (left), as described in Meterial and Methods (scale bar, 50 μm). **(C)** MitoSOX-stained H1299 and SiHa cells were exposed to 60 μM (50 μM in case of D54MG cells) DHA with 1 h of 5 mM NAC pretreatment. After 4 h of DHA exposure, the fluorescence of MitoSOX-stained cells were observed by fluorescence microscopy (scale bar, 50 μm). **(D)** DHA reduces MMP. PA-1 cells were stained with 25 nM TMRE, exposed to 5 mM NAC for 1 h and then DHA was added into the media followed by a further 4 h incubation. MMP was assayed by flow cytometry analysis (left). Right, data are presented as the average mean intensity fluorescence (MFI). ***, *P* < 0.001. Each bar represents the mean of three determinations repeated in three separate experiments. **(E)** Decrease in OCR by DHA treatment. PA-1 cells were seeded in 24-well XF analysis plates, and treated with 40 μM of DHA with 1 h of 5 mM NAC pretreatment. The OCR was monitored for 2 h, and calculated relatively to the vehicle control and average of five wells is shown.

ROS are potent regulators of MAPK activity [10,12], we therefore examined the potential involvement of ROS production in DHA-induced MAPKs activation. The effect of DHA on the production of superoxide was examined by monitoring DHE fluorescence. DHA treatment increased intracellular superoxide levels, and treatment with the antioxidant NAC blocked intracellular superoxide production in PA-1 cell line (Figure 3A). Since mitochondria are the main source of ROS in mammalian cells [11], we asked whether DHA-induced ROS were derived from mitochondria by measuring mitochondrial ROS production using the MitoSOX probes. The results (Figure 3B-C) showed that DHA enhanced the mitochondrial superoxide levels, and anoxidants NAC effectively blocked this effect of DHA, indicating that DHA induces ROS overproduction, in particular that of mitochondrial superoxide. Excessive mitochondrial ROS generation is associated with changes in mitochondrial function [22]. To ensure our above findings, and to determine whether the DHA-induced mitochondrial ROS is accompanied by mitochondrial dysfunction, we examined the MMP, which is an index of mitochondrial function [22], by labeling mitochondria with TMRE. As shown in Figure 3D, TMRE staining intensity decreased dramatically in response to DHA treatment. Furthermore, NAC treatment almost completely restored the decreases in TMRE intensity induced by DHA. The DHA-induced mitochondrial malfunction was further confirmed by measuring OCR (i.e., mitochondrial respiration rate). DHA remarkably decreased OCR, and NAC partially reversed this inhibitory effect of DHA (Figure 3E), suggesting that DHA-induced mitochondrial ROS production indeed impairs the function of mitochondria. Taken together, these results imply that mitochondrial ROS contributes to the increased level of cellular ROS induced by DHA.

### DHA-induced MAPKs activation is required for apoptosis

**Figure 4:**
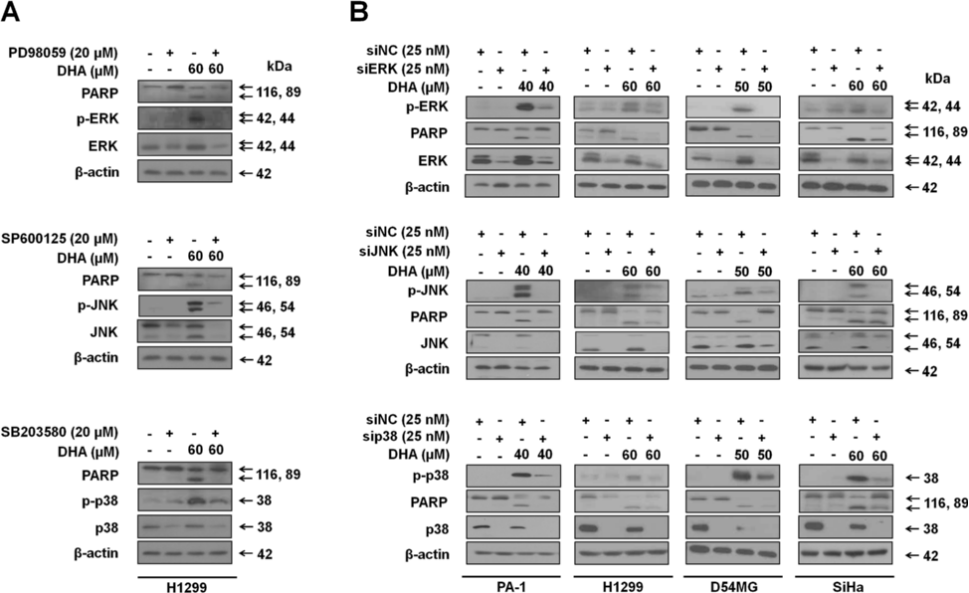
**Activation of MAPKs is responsible for the apoptosis induced by DHA. (A)** Indicated MAPKs inhibitors were added to H1299 cells 1 h before DHA treatment for 24 h. The protein levels of PARP and MAPKs were then examined by western blot. **(B)** Indicated cancer cells were treated with non-targeting control siRNA (siNC) or siRNAs specific for conventional MAPKs genes (siERK, siJNK and sip38). At 18 h after transfection, cells were incubated with the indicated doses of DHA for 24 h (12 h in cases of PA-1 cells). Then, cells were harvested and western analysis was performed using the following antibodies: PARP, MAPKs and actin. The data shown are representative of three independent experiments with similar results.

To unveil the role of MAPKs activation in DHA-induced apoptotic cell death, H1299 cells were first exposed to DHA in the absence or presence of the MAPK inhibitors PD98059, SP600125 and SB202190, specific for ERK, JNK and p38, respectively. The level of apoptosis was monitored by westernblotting using antibodies against PARP. As shown in Figure 4A, PD98059, SP600125 and SB202190 decreased the protein levels of cleaved PARP induced by DHA. These results suggest that the activation of conventional MAPKs is essential for DHA-induced apoptosis. The effects of the MAPKs on DHA-induced apoptosis were further examined by siRNA mediated knockdown of ERK, JNK and p38. Compared to cells treated with control siRNA, knockdown of three conventional MAPKs decreased the DHA-induced apoptosis in all four cell lines, as revealed by the level of cleaved PARP (Figure 4B), confirming that inactivation of the conventional MAPKs diminishes the DHA-dependent induction of apoptosis in cancer cells.

### DHA-induced ROS production is responsible for the MAPKs activation

**Figure 5:**
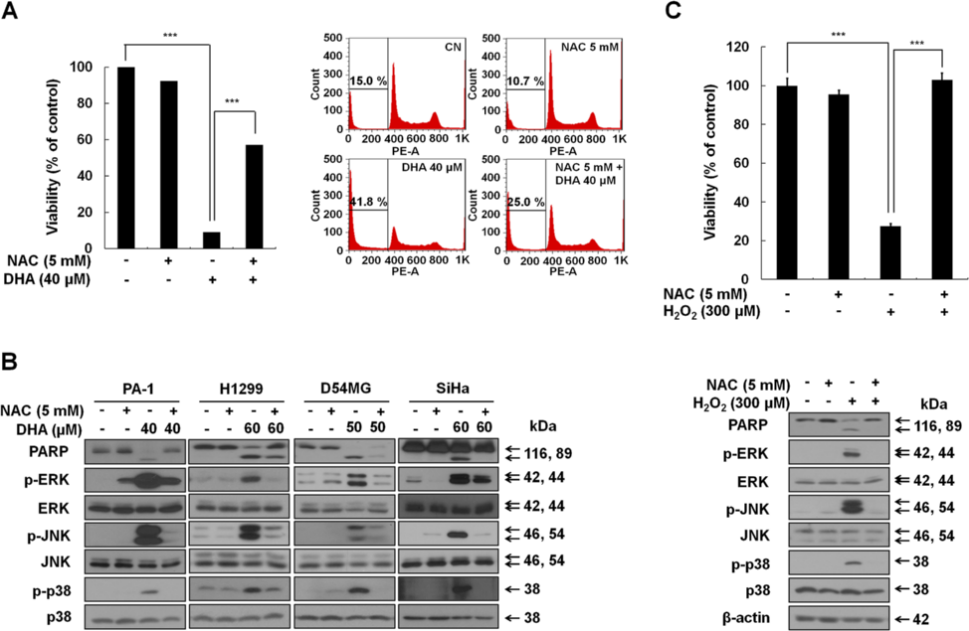
**Excessive ROS is associated with activation of MAPKs and subsequent apoptosis induced by DHA. (A)** DHA-induced ROS production is required for apoptosis. PA-1 cells were exposed to 40 μM DHA in the presence or absence of 5 mM NAC for 12 h. Left, cell viability was determined by the MTT assay. ***, *P* < 0.001. Each bar represents the mean of three determinations repeated in three separate experiments. Right, cells were collected to examine the percentage of cells in Sub-G1 phase by flow cytometry analysis. Samples were analyzed using FlowJo software. **(B)** NAC blocks the DHA-induced MAPKs activation. PA-1, H1299, D54MG and SiHa cell lines were incubated for 1 h with or without 5 mM NAC before exposure to the indicated doses of DHA for and 24 h (12 h in case of PA-1 cells). After cell lysis, PARP and MAPKs protein levels were examined by western blot analysis. **(C)** Apoptosis and MAPK activation in response to exogenous ROS, hydrogen peroxide. PA-1 cells were pretreated with or without 5 mM NAC for 1 h, followed by 300 μM hydrogen peroxide exposure for 12 h. Cell viability and the expression levels of cleaved PARP and MAPK were assessed by MTT assays (upper) and western blot analysis (lower). ***, *P* < 0.001.

Next, we sought to determine the relationship between excessive ROS generation and apoptotic cell death induced by DHA. To this end, PA-1 cells were first treated with 40 μM DHA in the presence and absence of NAC, and the levels of cell death were examined by MTT assays and flow cytometry. DHA dramatically decreased the number of viable cells (Figure 5A, left) and increased the Sub-G1 cell population (Figure 5A, right), which could be partially reversed by NAC, suggesting that DHA-induced apoptosis may be attributed to its capacity to trigger ROS overproduction. As our data suggested that the DHA-induced apoptosis was associated with excessive ROS production and MAPK activation, we investigated the possible link between apoptosis, ROS and MAPK. We found that the DHA-induced increases in cleaved PARP and phospho-MAPKs levels were remarkably attenuated by NAC pretreatment in all four tested cancer cell lines (Figure 5B). The effect of NAC on DHA-induced MAPKs activation was confirmed by immunocytochemistry assays. As shown in Additional file 3: Figure S3A-S3C, DHA increased both cytoplasmic and nuclear phospho-ERK, -JNK, and -p38 levels, whereas NAC reduced these effects of DHA. These data suggest that excessive cellular ROS accumulation contributes to the DHA-induced conventional MAPKs activation and apoptosis.

To verify the above findings, we used a different approach. PA-1 cells were first treated with exogenous ROS, H_2_O_2_, in the presence or absence of NAC. Then, cell viability and the levels of cleaved PARP and phospho-MAPKs were analyzed by MTT assays (Figure 5C, top) and western blotting (Figure 5C, bottom), respectively. H_2_O_2_ decreased cell viability and increased the expression levels of cleaved PARP as well as phospho-MAPKs; and NAC remarkably reversed these effects of H_2_O_2_. Furthermore, H_2_O_2_ also significantly increased the nuclear staining levels of phospho-ERK/JNK/p38, which could be prevented by NAC pretreatment (Additional file 3: Figure S3D-S3F). Together, these findings demonstrated that excessive ROS production is responsible for the activation of MAPKs, and that DHA-induced apoptosis is linked to the ROS-mediated MAPKs activation in cancer cells.

## Discussion

The ω3-PUFA, DHA prevents cancer through regulating multiple targets implicated in various stages of cancer progression, and one aspect of its antitumor effect involves inhibition of cell growth [1]. It has been shown that the growth-inhibitory effect of DHA is attributed to apoptosis and/or cell-cycle arrest, depending on the cell line studied [23,24]. In agreement with this, our results showed that the apoptosis induced by DHA is accompanied by cell-cycle arrest in H1299 and SiHa cells but not in PA-1 and D54MG cells. Although the identification of molecular determinant controlling either apoptosis or cell-cycle arrest as alternative modes of DHA-induced growth inhibition requires further investigation, these inconsistent observations indicate that detailed mechanistic events underlying the growth-inhibitory effect of DHA may be also cell type specific.

One major finding of this study is that the activation of conventional MAPKs (ERK, JNK and p38) is critical for the induction of apoptosis in tumor cells exposed to DHA. This finding confirms the results from previous studies [25-27], showing that DHA-induced apoptosis involves p38 activation. Meanwhile, it extends these studies by demonstrating that ERK and JNK activation is also required for the apoptosis in cells treated with DHA. The detailed mechanism by which activation of conventional MAPKs promotes DHA-induced apoptosis is still uncertain. We found that the apoptosis triggered by DHA was associated with altered protein levels of Bax and Bcl-2. Since conventional MAPKs activation has been shown to promote the expression and phosphorylation of pro-apoptotic Bax, and to disrupt anti-apoptotic Bcl-2 function, thereby resulting in apoptosis [20,28,29], it is reasonable to assume that Bax and Bcl-2 may act downstream of MAPKs activation to induce apoptosis in tumor cells treated with DHA. Notably, our data contrast with the findings of previous studies [30-33] which show that inactivation of ERK/p38 by DHA accounts for the apoptotic death of MCF-7, A549 and HCT-116 cancer cells. The reason for such disparate regulation of MAPKs activity in response to DHA is unclear, but might be related to the distinct genetic background (e.g., the prodeath or prosurvival role of basal MAPKs activity) of different types of cancer cells [13,14].

Previous studies suggest that the apoptosis inducing effect of DHA is at least partially attributed to its capacity to trigger mitochondrial ROS overproduction and malfunction [1,4,17,34]. Mitochondria are the major cellular organelles producing ROS and within mitochondria, the primary site of ROS generation is electron transport chain [11]. Therefore, our results that upon DHA exposure, the ROS, especially mitochondrial superoxide overproduced, and the OCR dramatically decreased with an increase in extracellular acidification rate (Figure 3 and data not shown), implying that DHA may cause a metabolic shift from oxidative phosphorylation to glycolysis and the disruption of electron transport chain.

Another question we addressed in the present study is the relationship between ROS, MARKs activation and apoptosis induced by DHA. ROS mediate MAPKs and the ROS-regulated ERK/JNK/p38 signaling in governing apoptosis under oxidative conditions have been widely investigated [10]. Although many studies have provided a general view that activation of the ERK pathway delivers a survival signal under oxidative stress, which counteracts the pro-apoptotic signaling associated with JNK and p38 activation [14], it is also reported that ROS-mediated ERK activation can induce apoptosis [15]. Our observations that DHA induced conventional MAPKs activation and apoptosis, which could be blocked by antioxidants are in agreement with the view that ROS-mediated activation of ERK/JNK/p38 in DHA-treated cancer cells is pro-apoptotic. Then, how do DHA-induced ROS result in the simultaneous activation of ERK/JNK/p38? One of potential molecules that may mediate this process is ASK1 (apoptosis signal-regulating kinase 1). ASK1 is substantially activated in response to a variety of ROS inducers, and has been shown to induce the activation of not only p38, but also ERK and JNK [35,36]. Thus, it is foreseen that DHA-induced ROS would simultaneously activate all three conventional MAPKs via upregulation of ASK1.

## Conclusions

**Figure 6:**
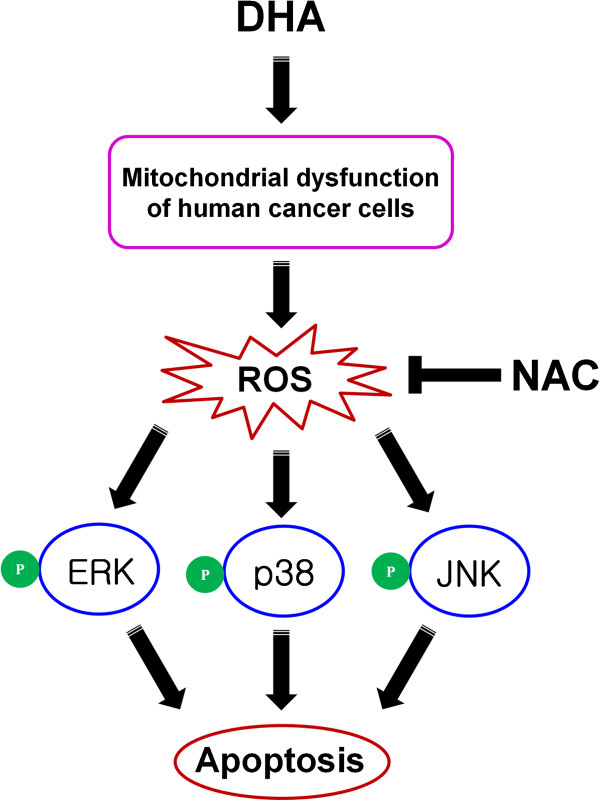
**Schematic model of DHA-induced apoptosis in human cancer cells.** The DHA-induced apoptosis was dependent on its ability to trigger excessive mitochondrial ROS generation and subsequent conventional MAPKs activation.

To summarize, the ω3-PUFA, DHA induces apoptotic cell death in various cancer cell lines. This increased apoptosis induced by DHA is dependent on its ability to trigger excessive mitochondrial ROS generation and subsequent conventional MAPKs activation (Figure 6). Thus, DHA may serve as an effective agent for the treatment and chemoprevention of human cancers.

## Abbreviations

ASK1: Apoptosis signal-regulating kinase 1; DHA: Docosahexaenoic acid; DHE: Dihydroethidium; DMEM: Dulbecco’s modified eagle medium; EPA: Eicosapentaenoic acid; ERK: Extracellular signal-regulated kinase; IAPs: Inhibitor of apoptosis proteins; JNK: c-jun N-terminal kinase; MAPKs: Mitogen-activated protein kinases; MEM: Minimum essential medium; MMP: Mitochondrial membrane potential; MTT: Thiazolyl Blue Tetrazolium Bromide; NAC: N-acetyl-L-cystein; OCR: Oxygen consumption rate; PUFA: Polyunsaturated fatty acid; ROS: Reactive oxygen species; siRNA: Small interfering RNA; TMRE: Tetramethylrhodamine, ethyl ester; TUNEL assays: Terminal deoxynucleotidyl transferase dUTP nick end labeling assays.

## Competing interests

The authors have declared no conflict of interest.

## Authors’ contributions

SJ, KJ, NK, SS, SK, KS Song, JYH, JHP, KS Seo, JH and KL participated in concept, design, data collection, data analysis, and data interpretation. GRK and SKP participated in concept and data interpretation. TW, JIP and KL participated in data interpretation and made supervision of the study. All authors have read and approved the final manuscript.

## Pre-publication history

The pre-publication history for this paper can be accessed here:

http://www.biomedcentral.com/1471-2407/14/481/prepub

## Supplementary Material

Additional file 1: Figure S1 DHA induces apoptosis. **(A)** DHA reduces cell viability in dose- and time dependent manner in PA-1, H1299, D54MG and SiHa cells. Cells were treated with the indicated doses of DHA for 0, 6, 12, 24 and 48 h. Cell viability was measured with the MTT assays as described in the Materials and Methods. IC_50_ values of DHA for four cell lines at exposure duration of 24 h were shown. Each bar represents the mean of three determinations repeated in three separate experiments. **(B)** DHA time-dependently induces apoptosis. PA-1 cells were treated with 40 μM DHA for the indicated time, and cleaved PARP as well as caspase-3 protein levels were detected by western blot analysis. Click here for file

Additional file 2: Figure S2 The growth-inhibitory effect of DHA is cell type specific. PA-1 **(A)**, H1299 **(B)** and SiHa **(C)** cells were exposed to increasing concentrations of DHA for 6, 12 and 24 h, and cell cycle was measured by FACS analysis. Samples were analyzed using FlowJo software. The data shown are representative of three independent experiments with similar results. Click here for file

Additional file 3: Figure S3 Generated ROS by DHA increases MAPKs activation. **(A-C)** PA-1 cells were first incubated with 5 mM NAC for 1 h; then indicated doses of DHA were added and the cells were incubated for 6 h. Cells were stained with antibodies against phospho-ERK **(A)**, phospho-JNK **(B)**, and phospho-p38 **(C)** and analyzed by the immunofluorescence assay (scale bar, 100 μm). **(D-F)** Hydrogen peroxide enhances MAPKs activation. PA-1 cells were first exposed to 5 mM NAC for 1 h; then 300 μM hydrogen peroxide was added and the cells were incubated for 6 h. Cells were immunofluorescently stained with antibodies against phospho-ERK **(D)**, phospho-JNK **(E)**, and phospho-p38 **(F)** (scale bar, 100 μm). Click here for file
