# Supplementary figures and images for: Docosahexaenoic acid-induced apoptosis is mediated by activation of mitogen-activated protein kinases in human cancer cells
Source: BMC Cancer. 2014 Jul 3;14:481. doi: 10.1186/1471-2407-14-481 (PMC4094407; doi:10.1186/1471-2407-14-481)

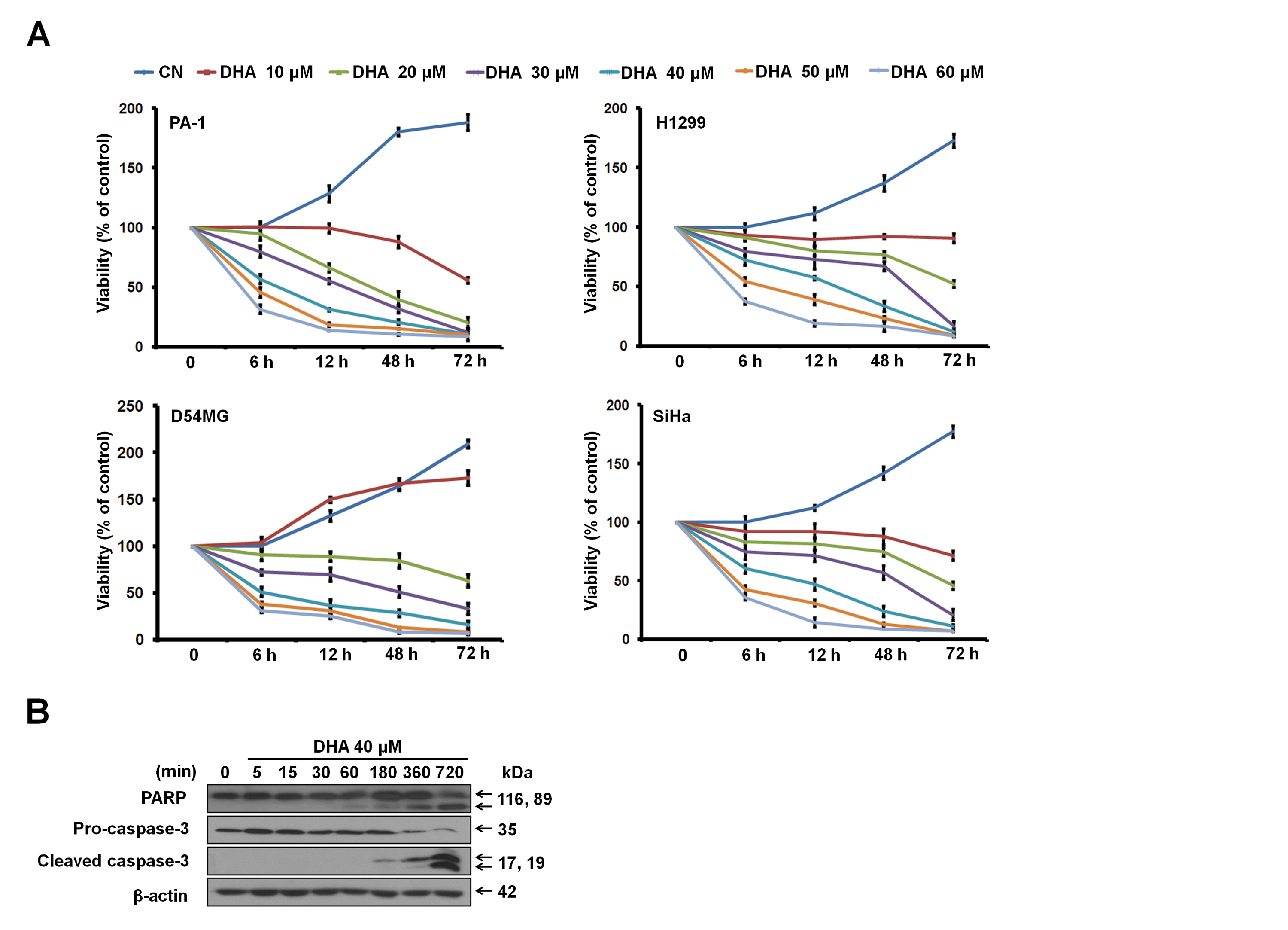

Supplement: Additional file 1: Figure S1 — DHA induces apoptosis. (A) DHA reduces cell viability in dose- and time dependent manner in PA-1, H1299, D54MG and SiHa cells. Cells were treated with the indicated doses of DHA for 0, 6, 12, 24 and 48 h. Cell viability was measured with the MTT assays as described in the Materials and Methods. IC50 values of DHA for four cell lines at exposure duration of 24 h were shown. Each bar represents the mean of three determinations repeated in three separate experiments. (B) DHA time-dependently induces apoptosis. PA-1 cells were treated with 40 μM DHA for the indicated time, and cleaved PARP as well as caspase-3 protein levels were detected by western blot analysis. [file 1471-2407-14-481-S1.tiff]

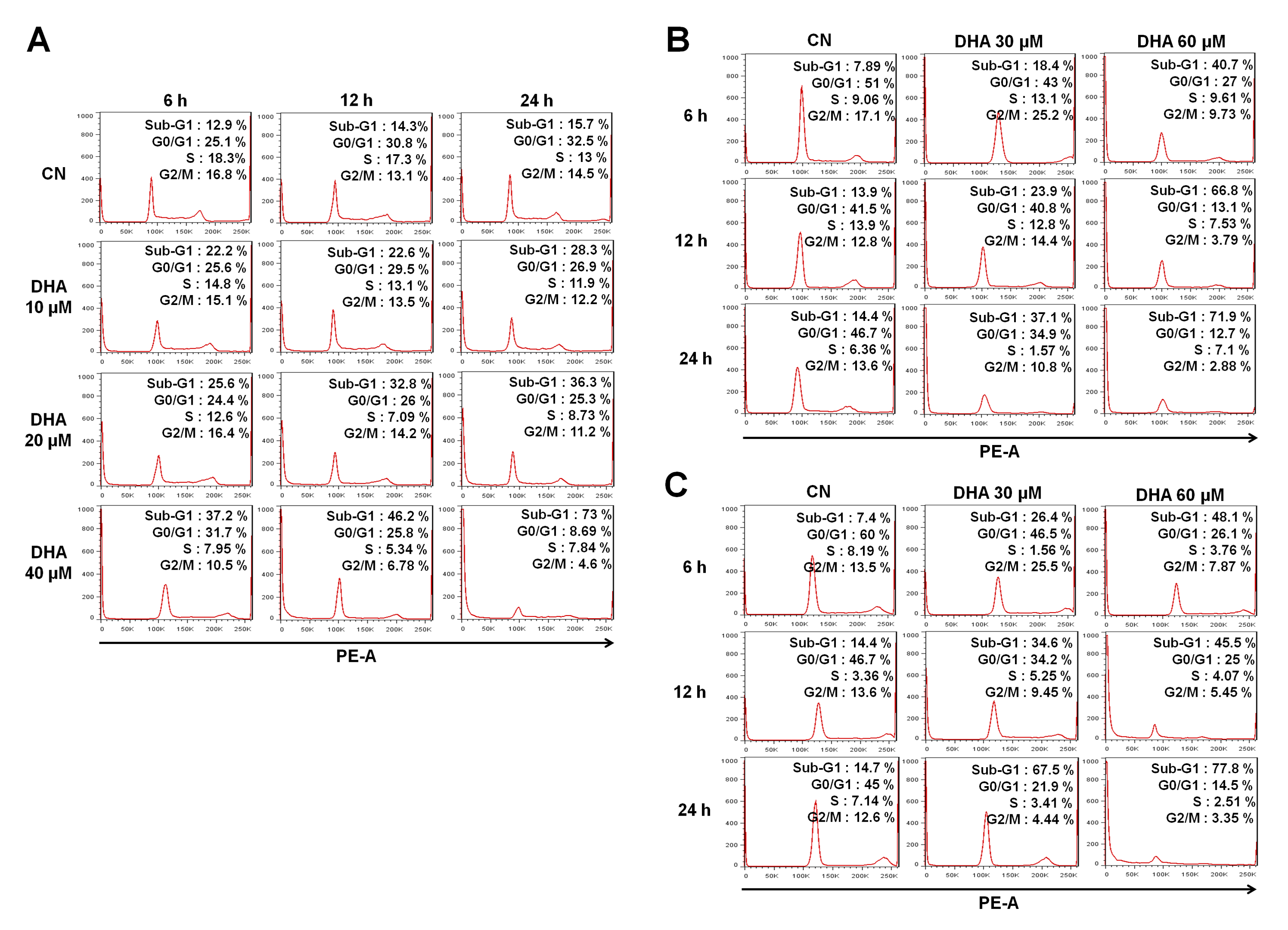

Supplement: Additional file 2: Figure S2 — The growth-inhibitory effect of DHA is cell type specific. PA-1 (A), H1299 (B) and SiHa (C) cells were exposed to increasing concentrations of DHA for 6, 12 and 24 h, and cell cycle was measured by FACS analysis. Samples were analyzed using FlowJo software. The data shown are representative of three independent experiments with similar results. [file 1471-2407-14-481-S2.tiff]

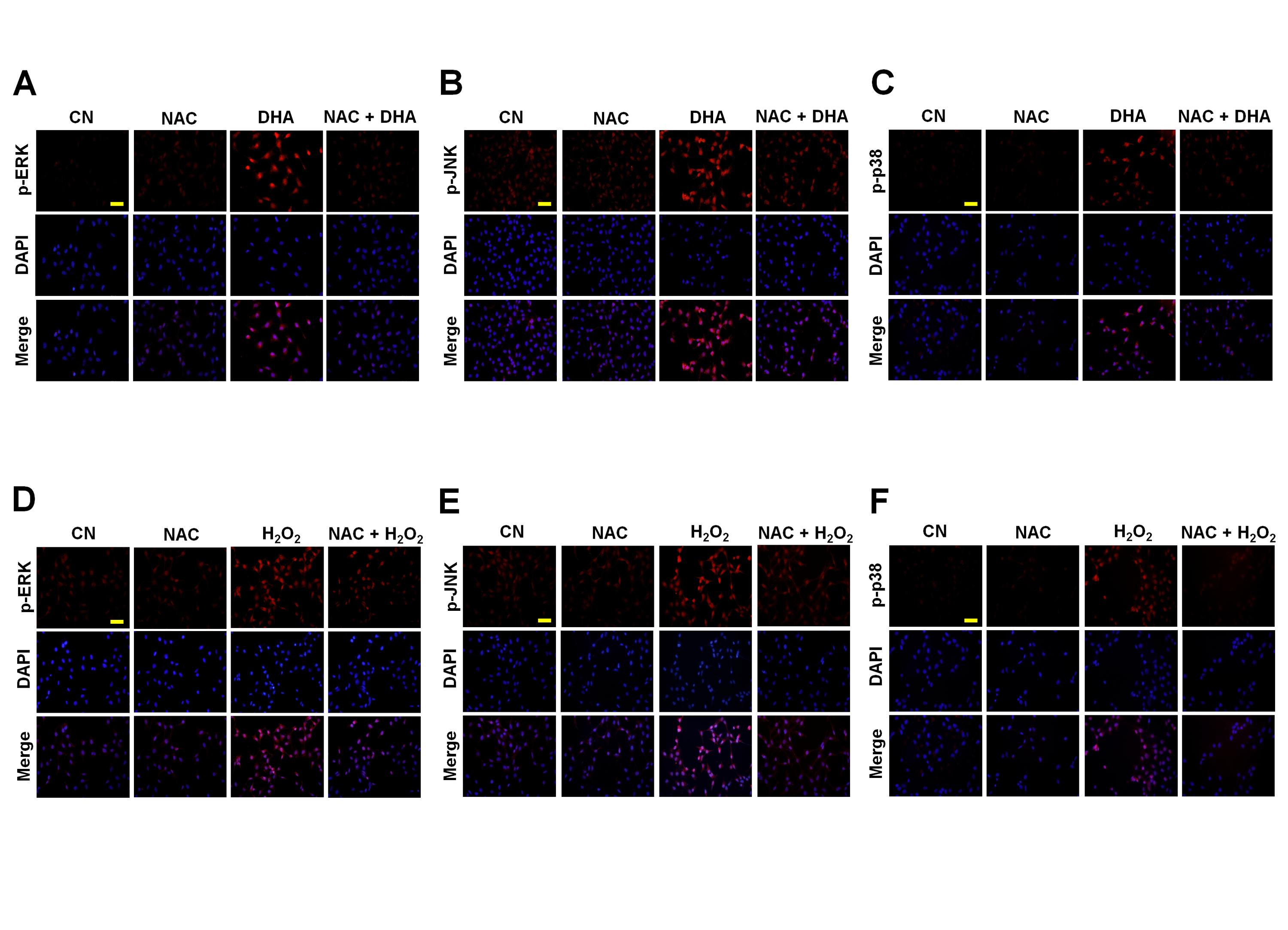

Supplement: Additional file 3: Figure S3 — Generated ROS by DHA increases MAPKs activation. (A-C) PA-1 cells were first incubated with 5 mM NAC for 1 h; then indicated doses of DHA were added and the cells were incubated for 6 h. Cells were stained with antibodies against phospho-ERK (A), phospho-JNK (B), and phospho-p38 (C) and analyzed by the immunofluorescence assay (scale bar, 100 μm). (D-F) Hydrogen peroxide enhances MAPKs activation. PA-1 cells were first exposed to 5 mM NAC for 1 h; then 300 μM hydrogen peroxide was added and the cells were incubated for 6 h. Cells were immunofluorescently stained with antibodies against phospho-ERK (D), phospho-JNK (E), and phospho-p38 (F) (scale bar, 100 μm). [file 1471-2407-14-481-S3.tiff]
